# Mechanical Properties and Microstructure Evolution of Al-Li Alloy Under the PHF Process

**DOI:** 10.3390/ma18030566

**Published:** 2025-01-26

**Authors:** Zhiang Gong, Xiang Huang, Peiliao Wang, Huijuan Ma

**Affiliations:** 1 Hubei Key Laboratory of Advanced Technology for Automotive Components, Wuhan University of Technology, Wuhan 430070, China; 286802@whut.edu.cn (Z.G.); 320028@whut.edu.cn (X.H.); wangpeiliao@126.com (P.W.); 2 Hubei Collaborative Innovation Center for Automotive Components Technology, Wuhan 430070, China; 3 Hubei Research Center for New Energy & Intelligent Connected Vehicle, Wuhan 430070, China; 4 Dongshi (Wuhan) Automotive Parts Co., Ltd., Wuhan 430070, China

**Keywords:** PHF, Al-Li alloy, phase transformation, microstructure regulation, mechanical properties

## Abstract

Currently, Al-Li alloys have been widely concerned in the aerospace and other fields due to their excellent comprehensive mechanical properties. However, the limitation of the long thermomechanical treatment still needs further improvement. Therefore, for an Al-Li alloy with multiple strengthening phases, this work proposes a pre-strain and pre-aged hardening warm forming (PHF) process. In the process, the multiphase precipitation and phase transformation are regulated by macro-control of the pre-strain, pre-aging, and warm forming stages. It is discovered that the 2A97 Al-Li alloy, with “7% pre-strain + 80 °C/16 h pre-aging + 250 °C/10 min warm forming”, exhibits the relatively optimal tensile/yield strength of 565.3 MPa/531.2 MPa. The addition of pre-strain facilitates the nucleation and precipitation of T_1_ phases through the consumption of δ′ phases and θ′ phases and promotes dynamic recrystallization during the warm forming process. The fine and uniform T_1_ phases are observed at the warm-maintaining time of 10 min. However, further extension of warm-maintaining time results in the coarsening of T_1_ phases and the reduction in strength. The proposed PHF process significantly shortens the thermomechanical treatment cycle of Al-Li alloys, which provides theoretical guidance for exploring the new short-process forming method.

## 1. Introduction

Lightweight aluminum alloys have long been a focus of interest in aerospace and other fields, as weight reduction remains a constant theme. Al-Li alloys have more prospects in terms of being lightweight compared to 2xxx and 7xxx aluminum alloys, because of the addition of Li further reducing the density of the aluminum alloy [1,2]. Meanwhile, the Al-Li alloys also exhibit significant advantages in high specific strength, high specific modulus, as well as good corrosion resistance and damage tolerance [3,4]. Therefore, Al-Li alloys have become a highly competitive type of lightweight aerospace materials for the future.

Al-Li alloys belong to the category of heat-treatable aluminum alloys. Artificial aging treatment is the most direct method to improve material strength, and strength enhancement has always been one of the primary development directions for Al-Li alloys. However, unlike the aerospace aluminum alloys such as 2219 and 7075, Al-Li alloys undergo multiphase precipitation and phase transformation during the aging, which leads to a highly complex aging process. For most Al-Li alloys, the main strengthening phases are δ’(Al_3_Li), θ’(Al_2_Cu), and T1(Al_2_CuLi). Among them, spherical δ′ phases are coherent with the aluminum matrix, while disc-shaped θ′ phases and T_1_ phases are semi-coherent with the aluminum matrix [5,6]. The T_1_ phases are considered to have the highest strengthening effect in most studies in terms of strength contribution [7,8]. Liu et al. [9] discovered that the 2A97 alloy, after 4% pre-strain and 48 h of cryogenic aging, exhibited excellent mechanical properties. The results revealed that at peak strength, the strengthening phases within the 2A97 Al-Li alloy predominantly consisted of fine and uniformly distributed T_1_ phases, with a small amount of δ′ phases also existing. Gao et al. [10] investigated the precipitation phase transformation of the 2A97 Al-Li alloy after friction stir welding and subsequent heat treatment. They also observed a large number of fine and dispersed T1 phases within the grains when the material reached its maximum strength. Furthermore, due to the different degrees of mismatch between the precipitated phases and the aluminum matrix, a certain precipitation sequence exists for the various phases [11]. Jo et al. [12] further clarified the precipitation sequence of the three main strengthening phases of 2A97 Al-Li alloy during artificial aging for different w(Cu)/w(Li) ratios as follows: SSSS → GP zones → δ′+θ′ → T_1_+δ′+θ′ → T_1_+δ′ → T_1_. Gayle et al. [13] and Howe et al. [14] suggested that in the early stages of aging, θ′ phases and T1 phases compete for Cu atoms, while δ′ phases and T_1_ phases compete for Li atoms. At the peak aging stage, T_1_ phases consume δ′ and θ′ phases to acquire Li and Cu atoms, respectively. However, during artificial aging, the slow dissolution of δ′ phases lead to the similarly slow coarsening rate of T_1_ phases, and results in the extended interaction process between δ′ and T_1_ phases [15,16]. Thus, it is evident that the multiphase precipitation and phase transformation occurring during the isothermal artificial aging process of Al-Li alloys result in the lengthy aging process. And the existing studies also show that single-stage artificial aging under T6 and T8 treatments for Al-Li alloys requires 48 h or more [9]. Li et al. [17] conducted heat treatment on 2050 Al-Li alloys with 16% pre-strain, and the mechanical properties met the component requirements after artificial aging at 150 °C/40 h. Therefore, further shortening the thermo-mechanical treatment cycle of the Al-Li alloys is an urgent issue that needs improvement. From the above studies, it is clear that the strength of the Al-Li alloys is closely related to the internal strengthening phases, which indicates that phase transformation is a key solution.

The same as 2xxx and 7xxx aluminum alloys, Al-Li alloys exhibit poor elongation and low formability at room temperature, significant anisotropy, large springback, and other problems, which make it challenging to meet precision requirements and result in its difficulty in forming complex-shaped components [18,19]. Various attempts have been made by researchers worldwide to achieve a more precise forming of Al-Li alloy components. Yang et al. [20] investigated the skin stretching technology of Al-Li alloys and discovered that defects such as surface “orange peel” and “slip lines” easily occurred during uniaxial tension. The defects were attributed to the non-uniform grain size (60–80 µm) within the material. Li et al. [21] applied creep aging forming on 2050 Al-Li alloy components and pointed out that the new generation of Al-Li alloys exhibits a unique five-stage “double initial stage creep characteristic” due to the combined effects of dislocations, precipitation, and solute atoms. For precise forming of complex-shaped components that are difficult to handle with conventional forming processes, superplastic forming is a key method. Jia et al. [22] discovered the effects of deformation temperature and strain rate on the superplastic deformation behavior of rolled 2A97 Al-Li alloy sheets. After the deformation temperature of 410 °C and the initial strain rate of 1 × 10^−3^ s^−1^, the material achieved a peak elongation of 791%. In the aerospace application, Al-Li alloy sheets are mostly used for forming thin-walled structures. Therefore, hot stamping and other sheet metal forming technologies remain widely applied. Gao et al. [23] formed complex-shaped 2060 Al-Li alloy components using the HFQ technology but only studied the effects of temperature on the forming properties of the Al-Li alloy, without addressing the effects of process parameters such as forming speed, lubrication conditions, and blank holder force. Fan et al. [24] investigated a thermoforming–quenching combined stamping process to form U-shaped bent parts from 2195 Al-Li alloys via high-temperature gas bulging and revealed the deformation and strengthening mechanisms. However, the above-mentioned forming methods require long artificial aging to ensure the strength of the Al-Li alloy components. To overcome the limitation, Hua et al. [25] proposed the pre-aged hardening warm forming (PHF) process. Through rapid short-term high-temperature solution treatment, some strengthening phases are retained. And the material is quickly transferred to a cold mold for high-temperature forming until the final paint baking treatment. The process effectively utilizes high-temperature plasticity while shortening the microstructure evolution process and ensuring the final mechanical properties of components. The PHF process, as a forming technique that regulates metastable phases to achieve desired mechanical properties, has already been applied to high-strength aluminum alloys such as 7075 [26], which provides a reference for improving and innovating Al-Li alloy forming methods. In summary, it is of great significance for promoting the application of the Al-Li alloys in aerospace to explore the combination of the 2A97 Al-Li alloy and PHF process. Moreover, it is also vital to elucidate the aging precipitation behavior and microstructure characteristics of the Al-Li alloy in the PHF process and reveal the microstructure evolution of the interaction between strengthening phases and dislocations under multiphase precipitation and phase transformation.

Thus, the study aims to investigate the effects of different PHF process parameters on the properties and microstructure of the 2A97 Al-Li alloy. By controlling pre-strain, pre-aging temperature/time, and warm maintaining temperature/time, the Al-Li alloys with different mechanical properties and microstructure evolution are obtained to analyze the phase transformation after pre-aging and warm forming. The microstructure characteristics and interactions between strengthening phases and dislocations under different process parameters are also investigated.

## 2. Materials and Methods

### 2.1. Materials

The materials used in the study are 2A97 Al-Li alloys, with 2.5 mm thickness H13-state. The 2A97 Al-Li alloy is the Gen3 novel Al-Li alloy developed in China, with good processing performance, and the precipitation sequence during artificial aging is as follows: SSSS → GP zones → δ′+θ′ → T_1_+δ′+θ′ → T_1_+δ′ → T_1_ [12,27]. The main chemical composition is shown in Table 1.

### 2.2. PHF Process

**Figure 1:**
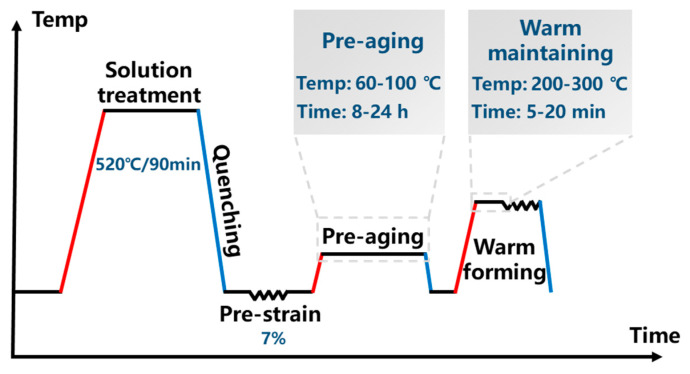
PHF process route of the 2A97 Al-Li alloy.

The 2A97 Al-Li alloy is subjected to heat treatment and warm forming under different process parameters. The specific process route is shown in Figure 1. The 2A97 Al-Li alloy is first solution-treated at 520 °C for 90 min. After the treatment, water quenching is performed at room temperature with a transfer time not exceeding 10 s to obtain solution-treated plates. Subsequently, a tensile deformation of 2.2 mm is applied along the rolling direction of the plates to correspond to 7% pre-strain. The deformation is based on the gauge length of the tensile sample, which is 31.8 mm. Then, pre-aging and warm maintaining are carried out in the constant-temperature drying oven at the different temperatures and times. The warm forming experiment is conducted on a CMT5205 microcomputer-controlled universal testing machine (MTS, Prairie, MN, USA). After the warm maintaining stage, the samples are rapidly transferred to the universal testing machine for a tensile deformation of 6% in the gauge section to simulate the deformation of the sheet during the warm forming process. The deformation amount is determined based on the forming simulation, which reveals that most regions experienced a strain of approximately 6%. The samples subjected to pre-strain are designated as Dx (x = 7). The samples pre-aged at 80 °C for different durations are designated as PAy (y = 8, 16, 24). The samples warm maintained at 250 °C for different durations are designated as WFz (z = 5, 10, 20).

### 2.3. Microstructure Characterization Methods

An electron backscatter diffraction (EBSD) test is conducted using a JSM-IT800 scanning electron microscope (SEM) (JEOL, Tokyo, Japan) equipped with an Oxford SYMMETRYS detector (Oxford, Oxford, UK). The samples are ground using sandpapers with roughness ranging from 400# to 2000#, followed by the mechanical polishing and electrolytic polishing. The electrolyte used for polishing consists of perchloric acid and ethanol in the 1:9 ratio. The polishing voltage is set at 25 V, with the electrolyte current of 0.5–1.5 mA, and the polishing time lasts 20–60 s. During observation, the scanning step size is set between 0.2 and 1 µm to analyze the grain transformation of the 2A97 Al-Li alloy after the warm forming.

The microstructure observations are characterized using an FEI Talos F200X transmission electron microscope (TEM) (JEOL, Tokyo, Japan) under the acceleration voltage of 200 kV. The TEM samples are first mechanically thinned to below 100 µm. A punch cutter is used to obtain the TEM samples with a diameter of 3 mm, which are further thinned using a Gatan 691 ion thinning instrument (Gatan, Pleasanton, CA, USA) to obtain the thin and clean areas. Additionally, the images during observation are collected from the <100>_Al_ and <110>_Al_ crystal zone axes, and the Fast Fourier Transform (FFT) and the Inverse Fast Fourier Transform (IFFT) are performed.

The differential scanning calorimetry (DSC) test is conducted using an STA449F3 thermo-gravimetric analyzer (NETZSCH, Germany). The DSC samples are obtained by the electrical discharge machining, with a diameter of 3 mm and a height of 1 mm. The tests are conducted in the high-purity N_2_ protective atmosphere, with a heating range of 50–500 °C and a heating rate of 10 °C/min. The evolution of strengthening phases is analyzed based on the differences in the heat-flow curves.

The X-ray diffraction (XRD) test is performed using an Empyrean X-ray diffraction instrument (PANalytical, Almelo, The Netherlands). The wavelength of the Cu-Kα radiation is set to 1.56 Å. The scanning speed is set to 2 °/min, and the scanning range is 10–90°. 

## 3. Results and Discussion

### 3.1. Precipitation Behavior of Strengthening Phases by the DSC Analysis

**Figure 2:**
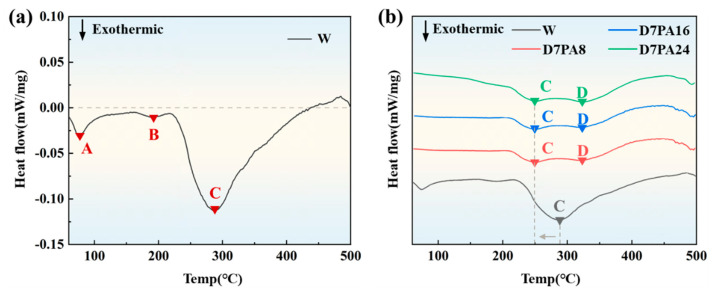
DSC curves of the 2A97 Al-Li alloy in different states: (**a**) solution-treated state(W-state); (**b**) with 7% pre-strain and different pre-aging times.

The pre-aging temperature and warm maintaining temperature are determined from the DSC curves of the 2A97 Al-Li alloy in different states, as shown in Figure 2. Along the negative y-axis direction, the exothermic peaks represent the precipitation of strengthening phases, while the endothermic peaks represent the dissolution of corresponding strengthening phases. As shown in Figure 2a, the DSC curve of solution-treated 2A97 Al-Li alloy exhibits three distinct exothermic peaks, designated A, B, and C. The exothermic peak A corresponds to the temperature with the highest precipitation efficiency of GP zones [28], approximately 80 °C. The exothermic peak B corresponds to the temperature with the highest precipitation efficiency of δ′ phases [29,30]. The exothermic peak C corresponds to the temperature with the highest precipitation efficiency of T_1_ phases [31,32], approximately 280 °C. However, the exothermic peaks A (GP zones) and B (δ′ phases) disappear in the DSC curve after 7% pre-strain and pre-aging (Figure 2b), which indicates that pre-aging at 80 °C can effectively promote the sufficient precipitation of GP zones and δ′ phases. In addition, Figure 2b shows that after pre-strain and pre-aging, the exothermic peak C, which corresponds to the temperature with the highest precipitation efficiency of T_1_ phases, shifts to a lower temperature compared to the solution-treated curve. Specifically, the temperature decreases from 280 °C to 250 °C. Therefore, to ensure the formation of more GP zones and δ′ phases during the pre-aging stage, and to promote the precipitation of T_1_ phases during the warm maintaining stage, 80 °C is chosen as the pre-aging temperature and 250 °C is determined as the warm maintaining temperature.

### 3.2. Mechanical Properties

**Figure 3:**
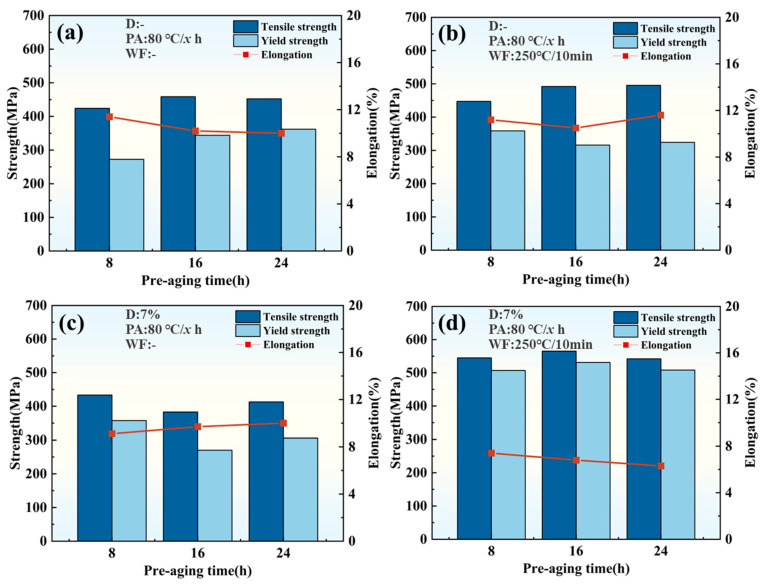
Mechanical properties of 2A97 Al-Li alloys under different process parameters: (**a**) without pre-strain, before warm maintaining; (**b**) without pre-strain, after warm maintaining; (**c**) with 7% pre-strain, before warm maintaining; and (**d**) with 7% pre-strain, after warm maintaining.

**Figure 4:**
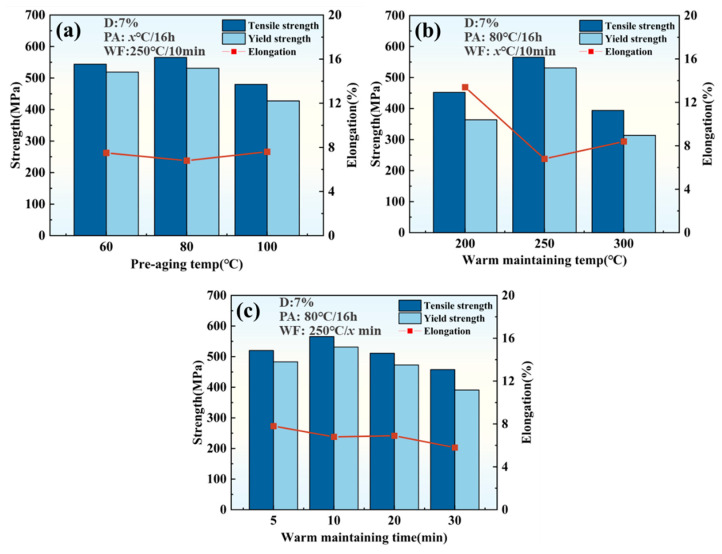
Effects of different process parameter variations on the mechanical properties of Al-Li alloys: (**a**) pre-aging temperature; (**b**) warm-maintaining temperature; and (**c**) warm-maintaining time.

To more accurately characterize the effects of pre-strain, pre-aging temperature/time, and warm maintaining temperature/time on the properties of 2A97 Al-Li alloys, four tensile samples are tested for each state. The average properties are summarized in Figure 3 and Figure 4.

Figure 3 shows the mechanical properties of 2A97 Al-Li alloys under different pre-aging times when the pre-aging temperature is 80 °C, and the warm-maintaining temperature/time is, respectively, 250 °C and 10 min. Without pre-strain, the tensile strength is relatively high after 16 h pre-aging. At 10 min warm maintaining, the tensile strength of each state increases by 33–53 MPa, but still remains below 500 MPa (Figure 3a,b).

With a 7% pre-strain, the strength of each state after pre-aging initially decreases and then increases as the pre-aging time extends. After 16 h pre-aging, the tensile/yield strength is only 383 MPa/270 MPa (Figure 3c). At 10 min warm maintaining, the mechanical properties of each state show the significant increase of 129.3–182.3 MPa. The tensile/yield strength of D7PA16WF10 reaches 565.3 MPa/531.2 MPa (Figure 3d), which is comparable to the mechanical properties obtained through the T6 and T84 treatments mentioned in the literature [9], but the aging cycle is shortened by 65–75% [32,33].

In addition, a series of mechanical property comparison tests are conducted for the different pre-aging temperatures, warm maintaining temperatures, and warm-maintaining times, as shown in Figure 4. The results show that for the PHF process, 80 °C, and 250 °C are relatively suitable temperature parameters for the pre-aging and warm-maintaining stages, respectively. And at 10 min warm maintaining, the tensile/yield strength is relatively optimal. However, with further extension of the warm-maintaining time, the mechanical properties of a 2A97 Al-Li alloy gradually decrease, which indicates that excessively long warm-maintaining time is detrimental to the strength improvement.

### 3.3. Dislocation Density by the XRD Analysis

**Figure 5:**
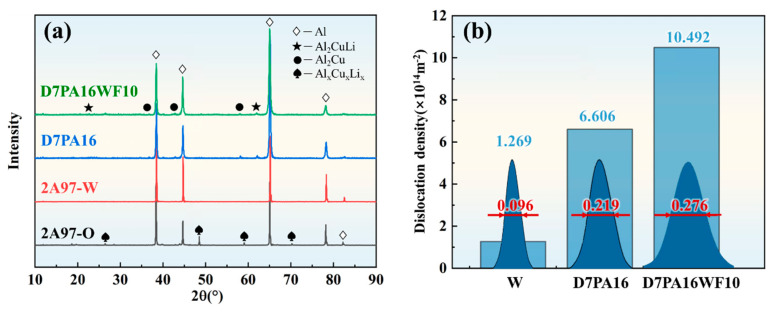
XRD analysis of 2A97 Al-Li alloys under different heat treatments: (**a**) XRD patterns; and (**b**) dislocation density at different stages.

Figure 5 shows the XRD analysis of 2A97 Al-Li alloys under the different heat treatments. In the annealed state (O-state), many characteristic peaks corresponding to the non-equilibrium phases Al_x_Cu_x_Li_x_ are observed in the microstructure of a 2A97 Al-Li alloy. After solution-quenching treatment at 520 °C for 1.5 h, the characteristic peaks disappear. And only the peak representing the aluminum matrix remains, which indicates a good degree of solution treatment. By comparing the XRD patterns before and after the warm forming, as shown in Figure 5a, it is discovered that the pre-aged 2A97 Al-Li alloy exhibits further formation of strengthening phases such as T_1_ phases and θ′ phases at the warm-maintaining temperature of 250 °C for 10 min.

In addition, the studies have indicated that the lattice imperfection can be reflected by the full width at half maxima (FWHM) of the diffraction peak of α-Al [34,35]. The modified Williamson–Hall method adopted is as follows [36]:

(1) $$\rho =\frac{B\cdot \varepsilon^{2}}{b^{2}}$$

In the equation, *ρ* represents the dislocation density, *ε* represents the microstrain, *b* is the Burgers vector of the aluminum alloy (*b* = 0.286 nm), and *B* is a constant related to the elastic modulus and the dislocation configuration of the material (*B* = 14.4).

As shown in Figure 5b, it can be observed that the dislocation density in the 2A97 Al-Li alloy increases significantly to 6.606 × 10^14^ m^−2^ after the 7% pre-strain and pre-aging treatment at 80 °C. Although subsequent short-term warm maintaining at 250 °C causes partial dislocation annihilation, the large deformation during the forming process further increases the dislocation density.

### 3.4. Microstructure Observation by the EBSD and TEM

**Figure 6:**
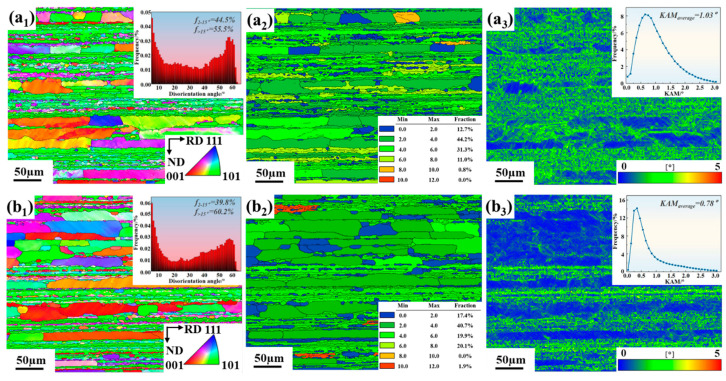
EBSD analysis of 2A97 Al-Li alloys without and with pre-strain: (**a**) without pre-strain; (**b**) with 7% pre-strain; (**a_1_**,**b_1_**) IPF analysis; (**a_2_**,**b_2_**) GOS analysis; and (**a_3_**,**b_3_**) KAM analysis.

In the PHF process, the forming of the Al-Li alloy sheets under the certain temperature conditions facilitates dynamic recovery or dynamic recrystallization. The microstructure undergoes the complex transformation during the warm forming process, which in turn affects macroscopic deformation behavior. Figure 6 shows the grain transformations in the 2A97 Al-Li alloy after the warm forming. It is observed that the grains are elongated, and the grain boundaries are generally serrated (Figure 6(a_1_,b_1_)). Upon further comparison of the differences without and with pre-strain, it is considered that the addition of pre-strain promotes dynamic recrystallization in the 2A97 Al-Li alloy during the warm forming process. It is also supported by the increased proportion of the high-angle grains (Figure 6(a_1_,b_1_)), the increase in the GOS recrystallized regions (Figure 6(a_2_,b_2_)), and the reduction in the Kernel Average Misorientation (KAM) values (Figure 6(a_3_,b_3_)).

**Figure 7:**
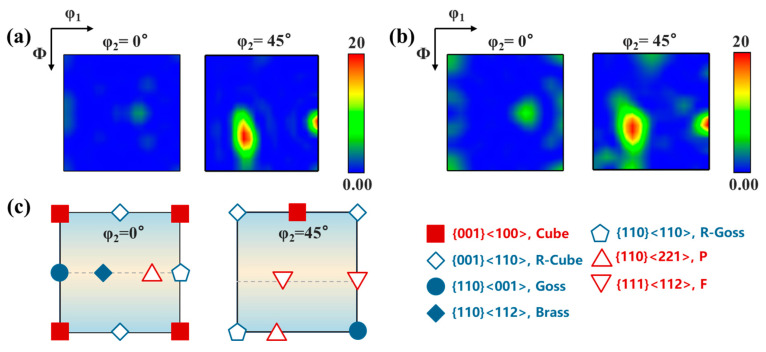
ODF observations of the PHF process without and with pre-strain: (**a**) without pre-strain; (**b**) with 7% pre-strain; and (**c**) ideal texture components in face-centered cubic metals.

Figure 7 indicates that the cube texture and the strengthening of the P texture appear with 7% pre-strain, which are the typical recrystallization textures [37,38].

**Figure 8:**
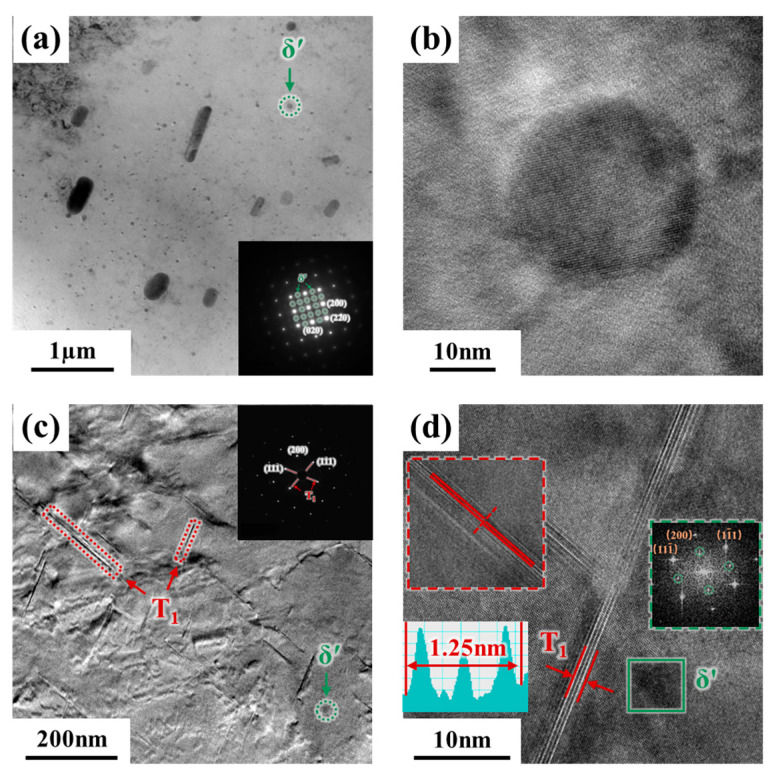
Observations of strengthening phases without and with pre-strain under pre-aging at 80 °C for 16 h: (**a**) PA16 strengthening phase distribution; (**b**) δ′ phase; (**c**) D7PA16 strengthening phase distribution; and (**d**) T_1_ and δ′ phases.

The transformation of strengthening phases is a key concern in the PHF process of the Al-Li alloys. Therefore, the microstructure of the 2A97 Al-Li alloy after the PHF process is observed and analyzed. Figure 8 shows the observations of strengthening phases without and with pre-strain under pre-aging at 80 °C for 16 h. The results indicate that without pre-strain, the strengthening phases of the 2A97 Al-Li alloy are mainly disk-shaped δ′ phases with a diameter of approximately 20 nm. With 7% pre-strain, the increased dislocation density accelerates the precipitation of T_1_ phases. The strengthening phases in the D7PA16 state are mainly δ′ phases and a small amount of T_1_ phases (Figure 8c). And compared to the state without pre-strain, the number of δ′ phases decreased. However, in the D7PA16 state, the thickness of T_1_ phases is approximately 1.25 nm, which is easily sheared by the dislocations and also observed in Figure 8d. The interacting mechanism for dislocations cutting through T_1_ phases within the range of thicknesses is verified by the study of Lin et al. [39].

**Figure 9:**
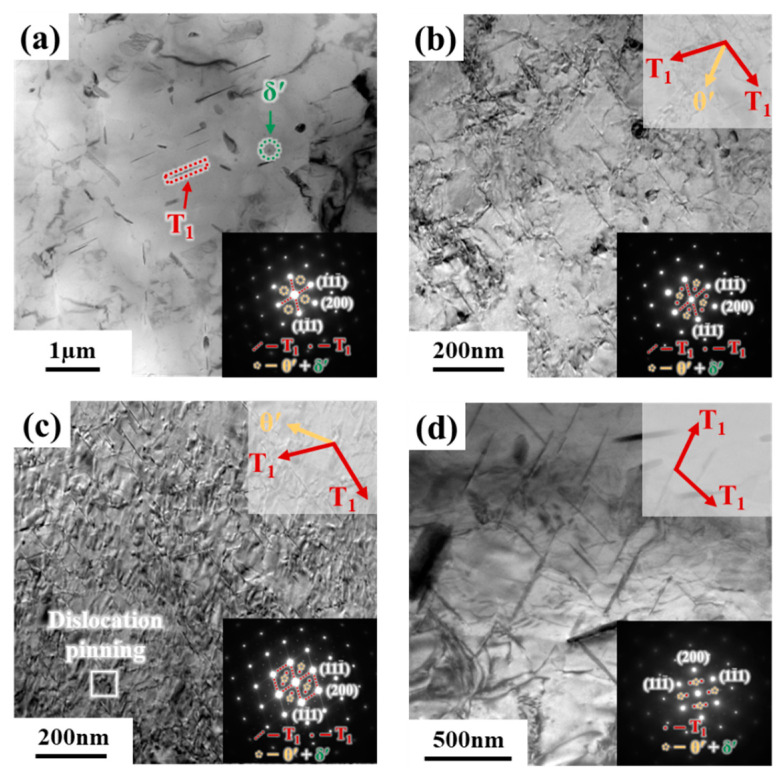
Observations of strengthening phases under different PHF process parameters for 2A97 Al-Li alloy: (**a**) PA16WF10; (**b**) D7PA16WF5; (**c**) D7PA16WF10; and (**d**) D7PA16WF20.

After the warm maintaining at 250 °C, T_1_ phases as the main strengthening phase nucleate and precipitate along the habit plane {111}_Al_. The diffraction characteristics of T_1_ phases are observed in selected area diffraction spots along the <110>_Al_ crystal zone axis (Figure 9a–d), and a small amount of θ′ phases and δ′ phases are also observed. The size of the main strengthening T_1_ phases without pre-strain are relatively larger. And it is observed that as the warm-maintaining time increases, the density of T_1_ phases initially increases and then decreases with 7% pre-strain. At 10 min, the T_1_ phases exist in a fine and dense distribution, and tend to nucleate near dislocations, as shown in Figure 9c. However, the T_1_ phases gradually coarsen, and the density decrease with the extended warm-maintaining time. Notably, a small amount of T_1_ phases precipitate during the pre-aging stage with pre-strain. Therefore, the new T_1_ phases form by consuming θ′ phases and δ′ phases, as well as the T_1_ phases grow and coarsen during the warm maintaining and are both observed after the warm forming. Consequently, as shown in Figure 9a, the overall size of T_1_ phases is relatively large, whereas T_1_ phases of varying sizes are observed in Figure 9b–d.

**Figure 10:**
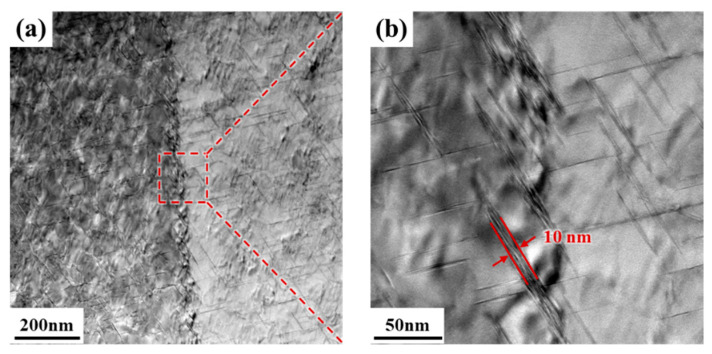
Observations of strengthening phases near grain boundaries in the D7PA16WF10 state. (**b**) is an enlargement of the area in the red box in (**a**).

In addition, the observation of strengthening phases near grain boundaries in the D7PA16WF10 state (Figure 10) demonstrates that the fine T_1_ phases near the grain boundaries are also uniformly dense, and there are no obvious precipitation-free zones (PFZ). The T_1_ phases on the grain boundaries are uniformly precipitated and exist in the form of multilayer stacking, with the thickness of the multilayer T_1_ phases approximately 10 nm. It is because the addition of pre-strain increases the dislocation density and induces T_1_ phases nucleation nearby, especially in the grain boundary/subgrain boundary region.

## 4. Conclusions

A pre-strain and pre-aged hardening warm forming (PHF) process for 2A97 Al-Li alloys is investigated in the study. The mechanical properties, dislocation characteristics, softening mechanism, multiphase precipitation, and phase transformation under the PHF process are clarified. The conclusions are as follows:

(1) Under the PHF process parameters of “7% pre-strain + 80 °C/16 h pre-aging + 250 °C/10 min warm forming”, the tensile strength and yield strength of the 2A97 Al-Li alloy reach 565.3 MPa and 531.2 MPa, respectively. The pre-aging time is reduced by 65–75% compared to the traditional artificial aging treatment.
(2) The addition of pre-strain promotes the transformation of low-angle grain boundaries into high-angle grain boundaries, as well as the reduction in the KAM values, the increase in the GOS recrystallized regions, and the appearance of two typical recrystallization textures: the cube texture and the strengthened P texture. It further increases the dislocation density and promotes the dynamic recrystallization during the warm forming process.
(3) The addition of pre-strain significantly accelerates the precipitation process and optimizes the distribution pattern. And the T_1_ phases nucleate and precipitate by consuming δ′ phases and θ′ phases during the warm maintaining. At the warm-maintaining time of 10 min, the T_1_ phases exist in a fine and dense distribution, which provides better pinning effects on dislocation slip and leads to optimal mechanical properties. However, further extension of the warm-maintaining time causes the T_1_ phases to grow and coarsen, resulting in a reduction in strength levels.

At present, the PHF process has been studied in conventional aluminum alloys, while research on Al-Li alloys, which are the advanced lightweight materials of aerospace and aeronautical equipment, remains in the exploratory stage. For instance, it would be worthwhile to conduct tests under complex stress conditions to simulate stamping forming, as well as to introduce machine learning algorithms for the optimization of mechanical performance and the efficient deduction of the process parameters.

## Figures and Tables

**Table 1:** Main chemical composition of the 2A97 Al-Li alloy.

Composition	Cu	Li	Mg	Zn	Mn	Fe	Si	Ti	Zr	Al
Content (wt %)	3.53	1.32	0.44	0.49	0.28	0.10	0.06	0.03	0.17	Balance

## Data Availability

The original contributions presented in this study are included in the article. Further inquiries can be directed to the corresponding author.
